# Sustaining small-scale fisheries through a nation-wide Territorial Use Rights in Fisheries system

**DOI:** 10.1371/journal.pone.0286739

**Published:** 2023-06-27

**Authors:** Eréndira Aceves-Bueno, Mateja Nenadovic, India Dove, Claire Atkins-Davis, Juan Salvador Aceves-Bueno, Abel Trejo-Ramirez, Cristina Rivas-Ochoa, Salvador Rodriguez-Van Dyck, Amy Hudson Weaver

**Affiliations:** 1 School of Marine and Environmental Affairs, University of Washington, Seattle, Washington, United States of America; 2 Nicholas School of the Environment, Duke University, Durham, North Carolina, United States of America; 3 Departamento de Historia, Universidad Autónoma de Baja California Sur, La Paz, Baja California Sur, México; 4 Mobula Conservation Project, La Paz, Baja California Sur, Mexico; 5 Data Analysis Human Intelligence, HUMINT, México DF, Mexico; 6 Sociedad de Historia Natural Niparaja, A.C., La Paz, Baja California Sur, Mexico; National Cheng Kung University, TAIWAN

## Abstract

Territorial Use rights in Fisheries (TURFs) are used around the world to manage small-scale fisheries and they’ve shown varying levels of success. Our understanding of what leads to different performance levels is limited due to several reasons. Firstly, these systems are often present in areas with low monitoring capacity where data is scarce. Secondly, past research has centered on the analysis of successful cases, with little attention paid to entire systems. Thirdly, research has been ahistorical, disconnected from the development process of TURF systems. Fourthly, TURFs are often viewed as homogenous ignoring the socio-ecological conditions under which they develop. To address these gaps, the study focuses on Mexico as a case study and context. The research first presents a historical overview of the development of TURF systems in Mexico, including the institutional and legal frameworks that have shaped their evolution. The paper then presents a TURF database that maps all TURF systems in Mexico, including their geographical locations and characteristics. In addition, the study presents case studies based on identified archetypes that showcase the diversity of TURF systems in Mexico, highlighting the different types of systems and the challenges they face. By presenting a comprehensive map of all TURF systems in Mexico, this research paper aims to make an important addition to the case studies in the global literature on TURF systems and provide a valuable resource for marine resource management policymakers, researchers, and practitioners.

## 1. Introduction

Small-scale fisheries (SSF; fishing activities that are carried out by individuals or communities, instead of companies, using low-technology gear) account for over 90 percent of the world’s commercial fishers and are responsible for some 40 percent of global fish catch [1]. As such they are critical to the livelihoods and food security of millions of people worldwide [2]. However, evidence suggests unregulated harvesting of marine species targeted by small-scale fishers can become a problem, with potential detrimental consequences on the livelihoods of coastal communities [3]. Some scholars argue these problems are associated with open access systems, where fishing grounds are available to large user groups that compete for resources until exhaustion (e.g. [4, 5]).

Solutions exist however, to encourage the sustainability of fisheries. For example, incentive-based management tools, which allow fishers to benefit from fishing while excluding others from those benefits through enforceable rights [6], whether species-based (i.e., catch shares / ITQs) or area-based (TURFs), have the potential to support the economic and ecological sustainability of fisheries [6–8]. One area-based management tool that has globally been used in small-scale or artisanal fishing communities is known as Territorial Use Rights in Fisheries (TURFs). This management tool falls within Other Effective Area-based Conservation Measures (OECMs) and specifically allocates one or more fishers exclusive access to a fishing territory in order to incentivize them to steward, not overexploit, marine resources and the environment [9, 10]. Therefore, TURFs are underpinned by secure property rights and as such can be considered a form of catch shares in which exclusivity in access to a portion of the fisheries catch is assigned spatially [8]. By securing access to the targeted resources, these systems allow the development of long-term harvest goals and reduce the race to fish [10–12].

Studies conducted in various countries such as Mexico, Brazil, Philippines, Indonesia, Japan, and Chile have found that TURFs exhibit significant variations in their design characteristics. These variations include temporary or permanent status, being allocations or true rights, the assignment to multiple or single species, and being standalone governance systems or part of umbrella organizations [12–20]. The studies have also shown that TURFs can have a positive impact not only on fish biomass but also on promoting social change towards more sustainable fisheries, and that increase in coastal resource stewardship can occur even before economic and ecological benefits are realized [12–18, 21]. TURFs have been particularly effective in promoting the recovery of benthic resources, as seen in the case of the Chilean loco [22, 23]. The Chilean TURF system has also played a significant role in enhancing the empowerment and social cohesion of coastal fishing organizations [24–29]. Lastly, TURFs have been a particularly important tool in allowing coastal communities and indigenous groups re-gain authority over the management of resources they depend on [30, 31]. As our need to better manage marine resources increase, studying these tools becomes critical. In particular, coastal regions with high population densities, where no take zones could impact food security, could benefit from TURFs and other OECMs.

While TURFs show promising results as a management tool, challenges remain such as the lack of support, enforcement capacity and compliance [27, 32–35]. Broader and international literature on TURFs, and generally small-scale fisheries, highlights the need for a more comprehensive and holistic approach to management [36]. Past research on the role and use of TURFs in small-scale fisheries has often been selective and limited in scope, focusing on specific species or sites (ex. [12–14, 48]). This selection bias can result in a lack of understanding of the broader social, economic, and environmental factors that influence performance [37]. Additionally, research on TURFs has often been ahistorical, with limited consideration given to the development processes that have led to their implementation, which substantially limits our understanding of such systems and hinders the efforts for their improvement. TURF systems are also rarely viewed in their entirety, with a focus on individual sites rather than the broader system. Furthermore, TURF systems are often viewed as homogenous entities rather than heterogeneous ones even though they vary significantly depending on the social, economic, and environmental contexts in which they are implemented. Therefore, a more nuanced understanding of TURFs is needed to assess their effectiveness and develop appropriate management strategies.

This paper aims to develop an analytical framework to help fill these knowledge gaps by exploring the Mexican TURF system. The study includes the history of small-scale fisheries development in Mexico, the TURF database, case studies illustrating the diversity of TURF systems, and insights into their effectiveness in improving fisheries management and the livelihoods of small-scale fishers. Furthermore, the categorization of the system into different archetypes enables the identification of potential areas for improvement and future research. While previous efforts focused on a few case studies, this study analyzes the Mexican TURF system in its entirety, providing a comprehensive view of its complexity, challenges, and opportunities for the improvement of artisanal fisheries management nationally and globally.

Our manuscript highlights the need for a more comprehensive and nuanced understanding of TURFs in the context of small-scale fisheries. By presenting a case study of Mexico, we show the importance of considering the broader social, economic, historical and environmental factors that can influence the performance of TURFs. The primary form of TURFs in Mexico are fishing concessions (we use the two terms interchangeably in the paper). The TURF database and case studies provide a fertile ground to study the diversity and effectiveness of TURF systems in achieving sustainable fisheries management. This paper’s findings can inform the development of more effective and holistic management strategies for small-scale fisheries worldwide.

## 2. Methods

### 2.1. Building a historical timeline and literature overview of Mexican TURFs

We conducted a literature search in Google Scholar, performed during June 10–18, 2022, using 7 search terms, which returned a total of 1,164 publications (S1 Table). Out of these, 404 publications contained all the relevant search terms as intended, with the exclusion of duplicate searches. We reviewed abstracts or introductions/conclusions when abstracts were not available to further identify publications in which concessions were either part of the study’s focus or were used to explain and/or support findings. We identified a total of 59 such publications, which we organized and evaluated based on year of publication, type of publication (peer reviewed journal, book, book chapter, etc.), and geographic and institutional focus state (S2 Table). We also used these publications to develop a historical timeline of concessions, better understand their origin and development, and in that way create a better understanding of the role they played in Mexican fisheries management.

### 2.2. Mapping the current Mexican TURFs landscape

In the fall of 2013, data on the locations of all Mexican concessions were requested through the system of Mexican transparency (INFOMEX). In January of 2014, over 200 printed documents containing concession titles and geographic coordinates of their respective territories were provided by the Mexican government. In 2021, a request for updated documents was made and a new set of documents was received. Our request was received by the INAI (request number 0819700021621) and redirected to CONAPESCA. We received photocopies of the titles of active concession territories (which include the geographic coordinates of the or polygons) between April 2000 and April 2020 and corroborated our previous map with the new information received. However, the old concession polygons were retained for analysis, since some concessions were in the process of being renewed. The difficulty in access to constantly updated data prompted us to create an interactive map (https://ereaceves.shinyapps.io/Tapp) where stakeholders can review the map, access our dataset and provide comments, which will allow the constant feedback of stakeholders familiar with the current state of Mexican TURFs.

The documents and coordinates for the Mexican concessions were systematically organized, reviewed, and summarized for analysis. Information for each concession included the title of the cooperative or name of the private owner, targeted species, year the concession was granted, duration of the concession, and the state in which the concession is located. The spatial territory of each concession was mapped using Google Earth and ArcPro and polygons were outputted for analysis in R software. Two-hundred and forty concessions were mapped, however, discrepancies in metadata or erroneous spatial information required the exclusion of several polygons from the map, which currently contains two-hundred and twenty-six polygons. Additional concession shapefiles were obtained from the literature [38] and mapped using QGIS georeferencing tool. Static maps and an interactive map were created in R programming software with the Leaflet package. Features of the interactive map that are dynamic include the ability to pan or zoom in or out across Mexico to see TURFs and when specifically hovering over a TURF with a mouse, the color of the TURF outline changes and the name of the fishing cooperative that owns the TURF.

We used the simple features (SF) R package to perform geometric measurements for each concession. The relative polygon *i* overlap was calculated as the number of overlapping polygons with polygon *i* divided by the maximum number of overlapping polygons in the country. The maximum number of overlapping polygons had a value of nine and was in the state of Sinaloa. The mean relative polygon overlap was then computed for each state and is presented in Table 1.

**Table 1:** Description of Mexican TURFs (i.e., concessions) in terms of their spatial distribution (per state), average size, and average species managed.

State	Concession titles	Concession polygons identified	Concession polygons mapped	Multi-resource concessions	Mean size km^2^*	Mean polygon overlap*	Mean species managed*
Baja California	10	54	33	3	39 (67)	0.06 (.07)	1.2 (0.6)
Baja California Sur	18	20	17	13	1,705 (1882)	0.16 (.09)	3.0 (1.9)
Chiapas	30	32	27	8	13 (16)	0.09 (.08)	1.7 (1.3)
Jalisco	1	1	1	1	3 (NA)	0 (NA)	3.0 (NA)
Sinaloa	122	124	98	0	30 (34)	0.24 (.19)	1.0 (0)
Sonora	3	10	10	0	17 (15)	0.30 (.17)	1.0 (0)
**Subtotal Pacific coast**	**184**	**234**	**186**	**25**	**--**	**--**	**--**
Quintana Roo	7	9	9	0	844 (588)	0.098 (.09)	1.0 (0)
Veracruz	19	19	14	12	58 (51)	0.11 (.09)	2.8 (1.5)
Yucatán	5	5	5	0	4,577 (2964)	0.08 (.09)	1.0 (0)
**Subtotal Atlantic coast**	**31**	**33**	**28**	**12**	**--**	**--**	**--**
**TOTAL**	**215**	**267**	**214**	**37**	**--**	**--**	**--**
*Mean (SD)							

### 2.3. Developing concession archetypes

**Fig 1:**
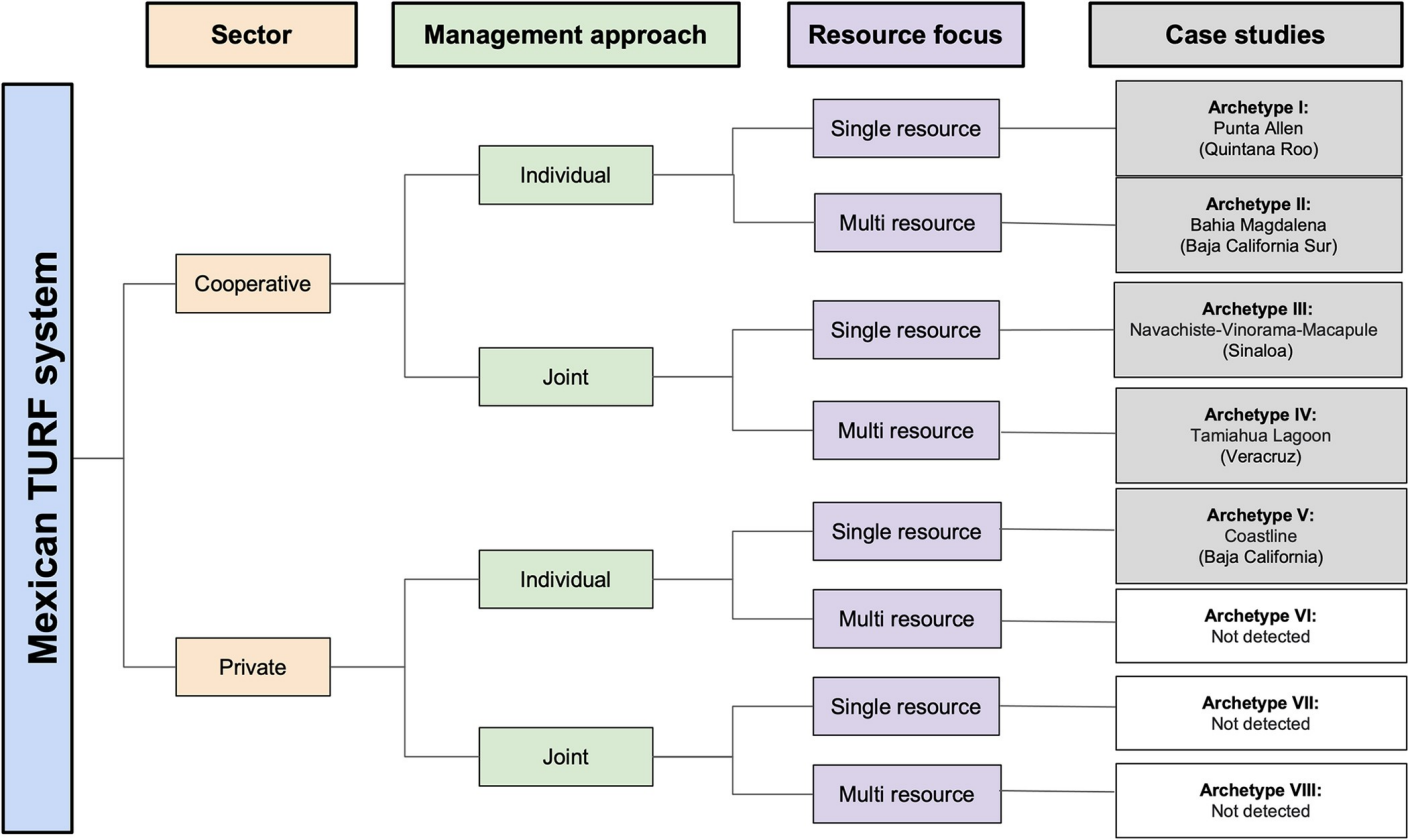
Categorization of concessions into different archetypes according to sector in charge, management structure in place, and resource focus.

**Fig 2:**
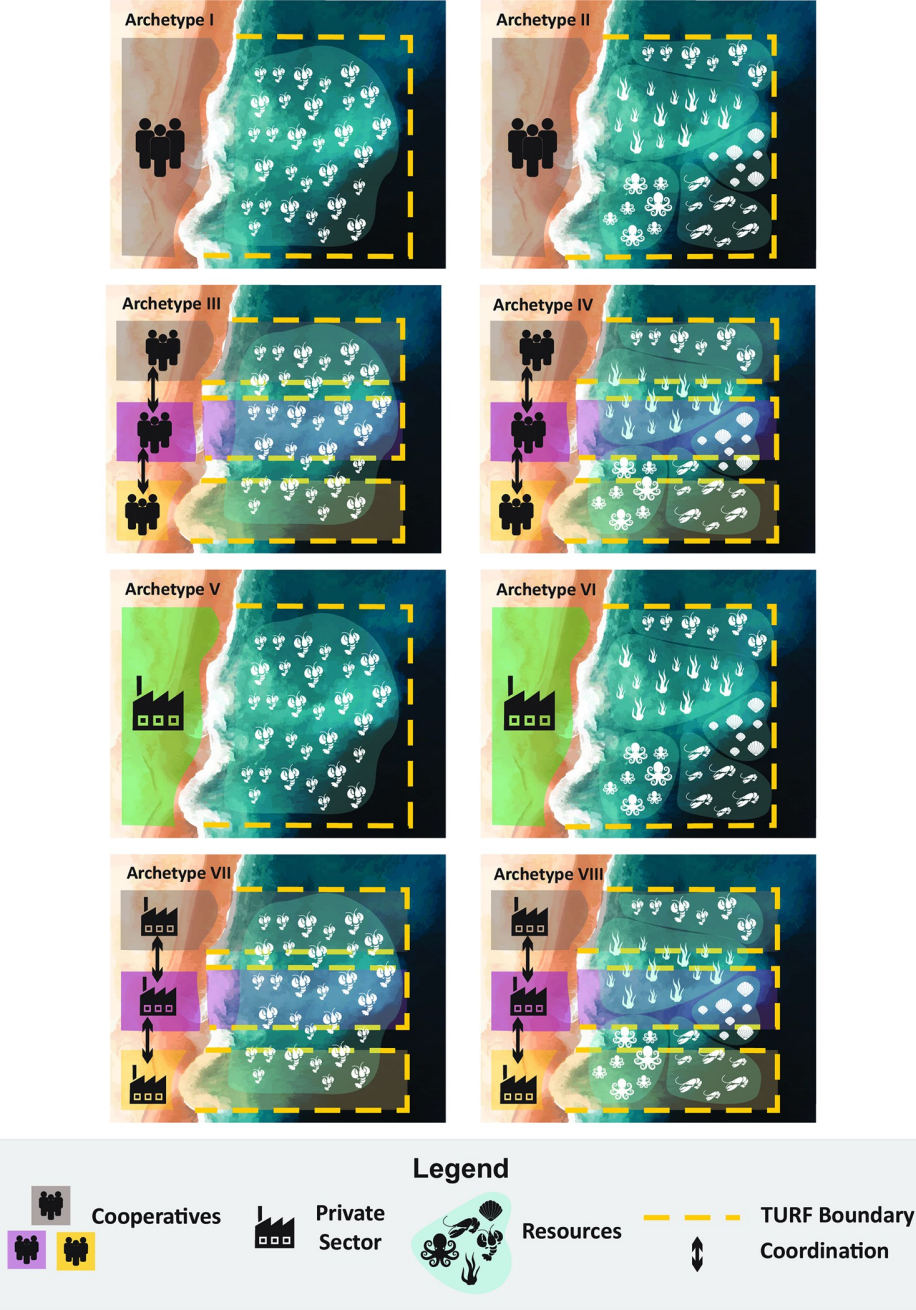
Eight archetypes of Mexican concessions.

Eight different archetypes of Mexican concessions were identified by grouping concessions according to the sector that received the concession as well as the concession’s management structure and resource focus (Figs 1 and 2). Sector separates the concession user into cooperative and private (business or individual permit holder) categories. Management structure indicates whether concessions are managed individually or jointly. Resource focus relates to the number of species managed with a concession and separates them into single resource and multi resource. We chose these three variables based on the empirical considerations from the literature and our own experiences given their influence in design and functionality of concessions in Mexico. These three variables also allow dissecting TURFs into more uniform groups that can be further reviewed in greater detail.

Out of the eight possible archetypes, we were able to identify five of them within our dataset. We then selected a case study to exemplify each existent archetype, namely Punta Allen in Quintana Roo for Archetype I, Bahia Magdalena in Baja California Sur for Archetype II, the Navachise-Vinorma-Macapule lagoon complex (NVM) for Archetype III, The Tamiahua lagoon complex for Archetype IV, and the sargassum TURFs of Baja California for Archetype V. We performed a case-study selection based on our long-term involvement (>20 years) in Mexican small-scale fisheries governance [11, 39–41]. Although the last three archetypes (VI-VIII) do not currently exist in Mexico, they are possible given the current regulatory framework and thus we considered necessary adding them to the archetype map.

A literature review was performed for all case studies to describe examples that fulfill each archetype of TURFs. In cases that were not well documented in the literature, information gathered from public sources was complemented by conversations with two key stakeholders. This was a particular need for the Navachiste-Vinorama-Macapule and the La Joya-Buenavista lagoon systems, for which we had conversations with local contacts. Information drawn from the literature and personal conversations included: a) a general description of the system (e.g., the ecosystem, main fisheries, current regulatory framework), b) description of the management structure (e.g., how is access determined? how are fishing activities coordinated?), and c) current challenges (e.g., state of the resources, observed conflicts, state of the governance system).

## 3. Results

### 3.1 Mexican TURFs in literature

Out of 59 analyzed publications, more than 50% were published after 2016, with the first recorded publication that discussed Mexican TURF/concessions occurring in 1998 (S2 Table). Majority of publications were in peer-reviewed journals (71%), master and doctoral thesis (11.9%), and book chapters (8.5%). In terms of geographic distribution, concessions were discussed in the context of six states: Baja California, Baja California Sur, Quintana Roo, Sinaloa, Sonora, and Yucatan. Out of these, the states of Baja California Sur (41.9%), Quintana Roo (24.2%), and Sonora (16.1%) were the most prevalent in literature, considering that some publications included more than one state. In cases where publications contained information that allowed us to specify a particular region of the state, two of the regions dominated the literature: the concessions of the Vizcaino desert (a.k.a. Pacifico Norte) region in Baja California Sur (35.9%) and Sian Ka’an Biosphere Reserve in Quintana Roo (30.8%).

### 3.2 History of Mexican TURFs

The history of Mexican fishing concessions over the last 150 years can be broadly divided into three periods, industrialist, socialist, and neoliberalist. Each period is characterized by an interplay of social, political, and economic forces at national and international levels.

During the industrialist period, which lasted from the late 19^th^ century to the early 20^th^ century, fishing concessions, a form of territorial concessions that were also applied to railways, mining, and oil, were seen as a policy tool used to attract national and foreign capital to a nascent state, as well as to colonize remote and inhospitable territories [42–45]. During this period, the principal focus of fishing concessions was the extraction of pearls along the coasts of the Baja California peninsula. A large foreign demand for pearls led to high profits, quickly attracting large investments needed to scale up harvesting equipment and infrastructure [46, 47]. The Porfirian government, eager to attract productive investments, provided institutional assurances in the form of concessions that would grant exclusive access to this resource [48]. In a period between 1884 and 1906 the federal government, through the Ministry of Development, issued 26 fishing concessions although only 10 of those were actively used [45, 46]. These concessions effectively enclosed the coast from Cabo San Lucas to the Colorado river delta and from Acapulco to the border with Guatemala [46]. The legal duration of individual concessions varied between 10 and 16 years with the possibility of renewal. Although the first concessions were granted to six companies formed by businessmen from Baja California, foreign capital quickly began to dominate the pearl mining, expanding its reach through a process of consolidation. By the end of the 19th century, pearl mining became a virtual monopoly in the hands of the British-owned Mangara Exploration Company Ltd., known locally as La Mangara [47].

The fall of the Porfiriato and the start of the Mexican Revolution in the early 20^th^ century mark the end of the industrialist period and a gradual move towards a socialist one, which lasted until mid 1980s. During this time, the importance of marine resources for the economic wellbeing of coastal communities and small-scale fishers was becoming more and more prominent [49]. The principal emphasis during this period was on strengthening social aspects of fisheries, mainly through the empowerment of worker collectives to which concessions played an important role [49, 50]. One of the first actions of the post-revolutionary government of President Francisco I. Madero was to revoke La Mangara’s concession on May 28, 1912. The two main reasons for Madero’s decision were a high level of dissatisfaction with the company’s practices and the need of independent fishers to access resources within the concession [47]. The initial government policy that affirmed the socialist focus in fisheries was the implementation of fishing zones, with preferential use reserved for the needs of adjacent coastal communities, which was codified in the first Fisheries Law of 1925 [49]. Over the next five decades, this trend would continue through the government’s active role in a promotion of fishing cooperatives as a preferred form of social organization, which was also used as a political instrument aimed at securing interests of a ruling party [49, 50]. With the implementation of the Fisheries Law of 1947 fishing cooperatives were granted exclusive access to some fishing resources including shrimp, oyster, abalone, lobster, totoaba, mullet, snook, and octopus, and were given federal support in the form of subsidies and infrastructure projects [49, 51].

The fishing concessions played an important role in this shift from private to social character, which was most visible in Sinaloa and Baja California peninsula. In the estuaries of southern Sinaloa early concessions during this period were given to individuals and private entities primarily for the exploitation of shrimp [50, 52]. The emergence of fishing cooperatives, which started in 1924 with the creation of the *La Unión de Pescadores de Escuinapa*, *S*.*C*.*L*. and consisting of 150 fishers, began the process of redistribution of concessions to social entities, enabled by federal authorities [50]. Over the next 50 years, an additional 27 cooperatives were formed in this region, many of which were granted concessions for the exploitation of shrimp [50]. The proliferation of cooperatives led to frictions and disputes among them, often due to the limitations in fishing activities posed by concessions, which resulted in changes of the spatial arrangement and boundary distribution of already established concessions (re-parcellation).

Along the Baja California peninsula, the first concession of the post-revolutionary regime was granted to a Japanese company for the exclusive extraction of abalone that stretched from the border with the USA to Magdalena Bay [53]. Its operations over the next 20 years led to the emergence of several fishing communities along this stretch of the coast. When the company went bankrupt in early 1930 as a result of the economic crisis associated with the Great Depression, local fishers continued to engage in the fishery and over time organized their activities in the form of cooperatives [53]. The Vizcaino desert region (i.e., Pacifico Norte) was the epicenter of the newly formed cooperatives, with the first one being the *California de San Ignacio S*.*C*.*L*., formed by 45 members in April of 1939 (California de San Ignacio S.C.L. 2017). Over the next 10 years, an additional five cooperatives were formed, each one receiving a fishing concession [54].

The gradual opening and alignment of Mexican politics with the free-market policies, which started in the mid 1980s, marked the end of the socialist period and a transition towards a neoliberalist one, which is still ongoing. The key aspect of this period is implementation of structural reforms aimed at deregulation of the markets, elimination of trade barriers, and domination of private capital [55]. With the publication of the Fisheries Law of 1992, the overall goal was to make fishing more efficient and to attract private investments [52]. To achieve this, cooperatives lost preferential treatment, which included elimination of their exclusive right of capture for shrimp, oyster, abalone, lobster, totoaba, mullet, snook, and octopus. According to our review of the official concession titles, the largest boom of fishing concessions occurred in the period between 1994 and 1999 during which some 170 concessions were granted. One explanation for this sudden rise is that many cooperatives sought concessions to secure access to the historically preferential resources which would allow them to increase competitiveness in the free market economy.

In general, and according to the current Mexican Fisheries Law [56], concessions can be given to a natural (i.e., individual fisher) or a juridical (e.g., cooperative, business) person. Each concession can be issued for one or multiple species, and a legal entity may hold more than one concession. Duration of concessions varies, although most of them are issued for 20 years and can be renewed.

### 3.3 Mapping the current Mexican TURFs landscape

**Fig 3:**
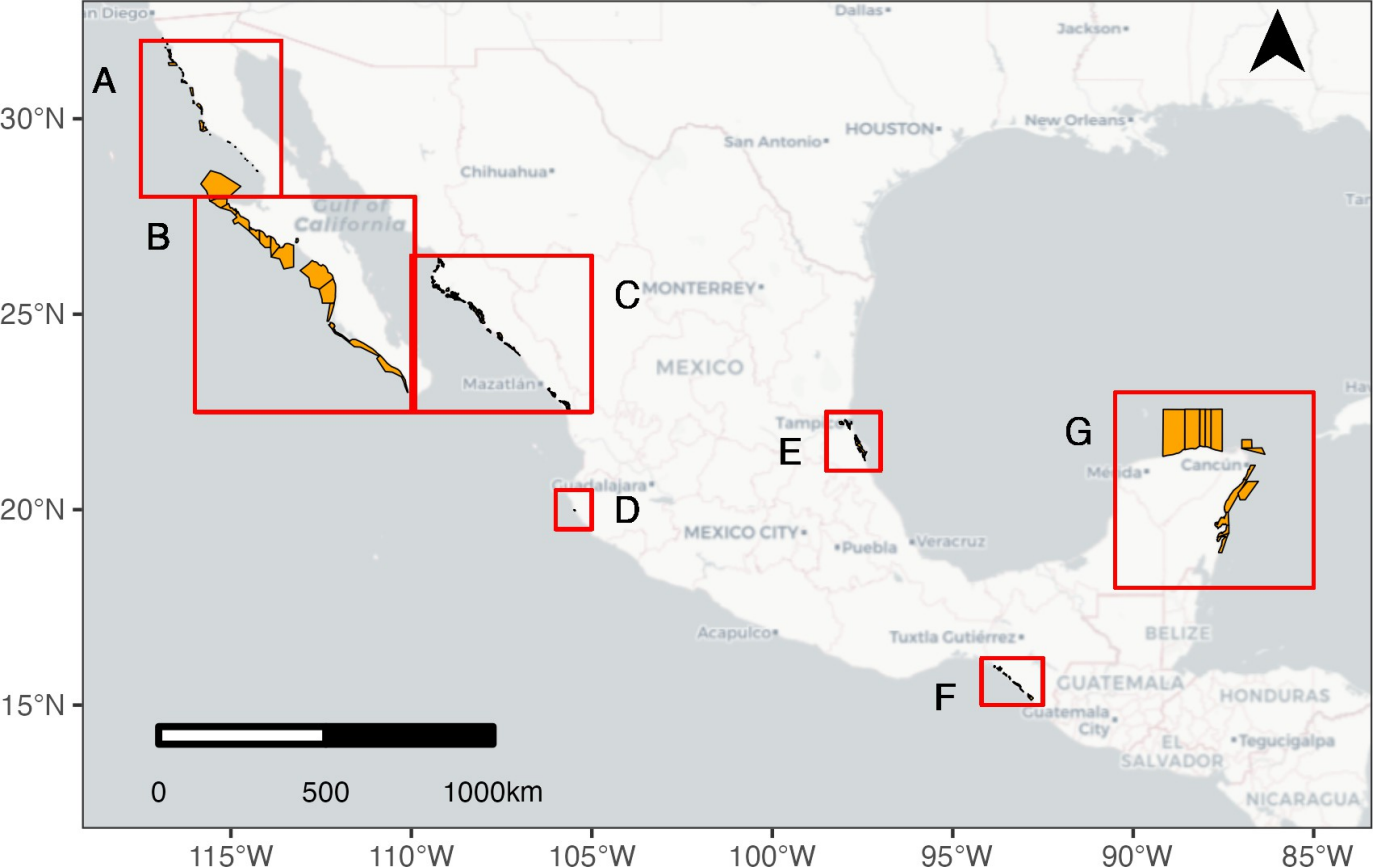
Mexican concession polygons mapped in this effort. The red squares highlight areas where concessions have been observed: A) Baja California Norte, B) Baja California Sur, C) Sinaloa/Sonora, D) Jalisco, E) Veracruz, F) Chiapas, G) Yucatan Peninsula. For a detailed and interactive map of Mexican concessions please visit https://ereaceves.shinyapps.io/Tapp.

We created static maps, an interactive map, and a summary table with information for 209 concessions and 206 concession holders (Fig 3, Table 1). All of the concessions are clustered among the six regions (Fig 3). Most concession holders (97.1%) held a single concession title and the majority of concession titles (89.8%) consisted of one concession polygon, with the marked exception of the titles in Baja California where one of the concessions for marine algae consisted of 38 polygons. Out of 206 concession holders, only three (1.5%) were private entities while the rest were cooperatives.

Overall, we were able to map 87.6% of all identified concession polygons (Fig 3, Table 1). Mapping revealed distinct patterns regarding the distribution, size, taxa, location of concessions and polygon overlap. Geographic distribution of concessions indicated that out of 17 states with a coastline, about half—nine states—have concessions in their waters. Some 87% of concessions were located along the Pacific coastline, with the majority found in the state of Sinaloa (57%) (Fig 3, Table 1). Another distinct feature that emerged from the mapping effort revealed that large parts of the coastline are lacking concessions. For example, within the Gulf of California, the states of Baja California Sur, Baja California, and most of Sonora have no concessions at all.

In terms of location, concessions can be separated into oceanic and estuarine. Majority of the concessions, some 66%, are estuarine and they are primarily located along the coasts of Sinaloa, Sonora, Chiapas, and Veracruz (Fig 3, panels C, D, and E). Concessions along the coast of Baja California peninsula and the Yucatan Peninsula are mainly located in open water (Fig 3, panels A, B, and F).

There were 26 taxa for which concessions were issued (S3 Table). All of these taxa, except for finfish, were benthic. Furthermore, six out of 25 benthic taxa were sessile. The greatest number of concessions were issued for shrimp (86.9%) lobster (13.1%), finfish (8.8%), and abalone (6.1%). Despite the diversity of species managed under concessions, the majority (82%) of them were for single species. Concessions in Quintana Roo, Sinaloa, Sonora, and Yucatan were exclusively single species while concessions in Baja California Sur were the most diverse, with an average of 4.2 species per concession (Table 1).

The average size of concessions is 424 km^2^, but the range substantially varies (0.04–9,110.84 km^2^). On average, the largest concessions were issued to cooperatives and found in Baja California Sur, Quintana Roo, and Yucatan while the smallest ones were located in Sonora, Sinaloa, Chiapas, Veracruz, Jalisco, and Baja California Norte. The smallest concessions were assigned to shrimp and fish in estuarine waters, as well as for algae harvesting.

### 3.4. Archetypes of Mexican TURFs

For its analysis, the Mexican TURF system can be categorized according to the sector in charge, the concession’s management system and the number of resources managed (Fig 1). This categorization allowed us to envision eight archetypes that could exist under the current regulatory framework, five of which are currently present in the country (Fig 2). It is important to highlight that the management of multiple resources can be achieved through a single or multiple concession titles. Archetype I comprise a concession that is given to a single cooperative and is permitted to harvest only a single species or species group, such as abalone, lobster, or shrimp. Archetype II is a concession also given to a single cooperative but is allowed to harvest multiple marine species within the exclusive fishing territory. Archetypes III and IV include concessions that are jointly managed among two or more cooperatives either for a single species or multiple species. Lastly, Archetype V consists of a concession given to a private company or person for a single species. Although we could not find evidence from the literature or from concession documents on the existence of Archetypes VI-VIII, which describe concessions given to a private person or company for multiple species or with joint management, these are possible under the current Mexican regulatory framework. This finding suggests a limited role of private persons or companies within the Mexican concession system.

Five case studies were chosen to exemplify each existing archetype, namely Punta Allen in Quintana Roo for Archetype I, Bahia Magdalena in Baja California Sur for Archetype II, the Navachise-Vinorama-Macapule for Archetype III, the Tamiahua lagoon complex for Archetype IV, and Agarmex for Archetype V.

### 3.5 Case studies

#### 3.5.1 Archetype I: Concession of *Vigía Chico fishing cooperative*

The concession of Vigia Chico is owned by a single cooperative, individually managed, and with a focus on a single species. It is located in the southeast of Mexico in Bahía de la Ascensión, in the state of Quintana Roo. The territorial boundaries defining the concession are within the Sian Ka’an Biosphere Reserve [57]. The bay is relatively large (760 km^2^) and shallow (average depth 3.5 m and maximum depth 7 m), covered by a coral reef, and surrounded by estuarine vegetation [58]. Meadows of mixed sea grasses and macroalgae provide necessary habitat for settlement of juvenile spiny lobster (*Panulirus argus*) [58]. The estuary, in turn, functions as a nursery ground where spiny lobster eventually recruit to the harvestable stock along the coral reef in adult stages [58]. This area is also secluded, and, along the entirety of the Bahia de la Ascension, there is only one small fishing village, which has roughly 500 to 600 people [15, 58].

Specifically, the Vigia Chico Cooperative, founded in 1968, is composed of about 80 fishers who use 55 small fishing boats [15, 57]. The only target species permitted to be harvested by cooperative members within their concession is the Caribbean spiny lobster (*Panulirus argus*). In Bahia de la Ascension and Bahia Espiritu Santo, the principal fishing gear was first introduced in 1960s and consists of a system of artificial habitats called “casitas” where lobsters aggregate [15, 59]. Aside from using casitas, the cooperative members have established individual fishing zones called *campos* that have a 25-meter, no-take buffer area between each campo [15].

There is evidence of a strong collective action within the cooperative for managing the sustainability of this fishery. The cooperative provides the processing infrastructure, access to market and marketing tools and covers enforcement and monitoring costs for the concession [15, 59]. Decisions are made through democratic processes involving all its members and include aspects of monitoring and enforcement. For example, the self-organizing nature of the cooperative was developed by local fishers themselves [59] and the surveillance system within the Sian Ka’an Reserve is strengthened since local fishers have the capacity to create rules and have independence from the government (Ex. [57]). Particularly important design features to note for this cooperative and concession include the 20-year tenure that has a strong likelihood of renewal, “clearly defined co-management responsibilities between the federal government and the Cooperatives,” and the use of individual marine plots, or *campos*, to maintain the accountability of members [15].

Overall, this system has allowed cooperative members to effectively perform a highly selective fishery through free diving to hand retrieve lobster (scuba and hookah are prohibited) facilitating the compliance with minimum size limits, protecting reproductive females, and maintaining a high quality of their products [59, 60]. Majority of the catch is sold internationally [61]. Since 1982, the Vigia Chico cooperative has been known to be the most productive fishing cooperative for lobster in the Mexican Caribbean [46]. The traditional fishing practices, regulations and efficient operation of the cooperative have allowed this fishery to become a model for sustainability and to obtain the Marine Stewardship Council certification [15].

Despite significant management efforts, the Vigia Chico Cooperative continues to encounter significant environmental and social challenges. In addition to dealing with the impact of climate change on their fishery, the cooperative is periodically subjected to the effects of hurricanes, which can alter the ecosystem and result in damages to fishers’ property and the surrounding villages, resulting in significant costs [57]. Mendez-Medina [57] also pointed out that the cooperative faces considerable challenges due to inadequate government support for monitoring and enforcement.

#### 3.5.2 Archetype II: *Concession of Bahia Magdalena fishing cooperative*

The concession of the Bahia Magdalena fishing cooperative is located within the Bahia Magdalena lagoon complex (BMLC) which is a highly productive system of along the Pacific coast of Baja California Sur [62]. Bahia Magdalena acts as critical nursery habitat and feeding grounds for many commercially important species [63]. Given its high productivity, it is the most important fishing region in Baja California Sur with a production around 60% of the total annual catch for the state (the second most productive of the country), primarily from industrial catches of sardines and tuna [62, 64].

The Bahia Magdalena fishing cooperative, founded in 1952, has a concession that grants it exclusive access to capture abalone (*Haliotis* spp.) and red lobster (*Panulirus interruptus*). Its 58 members use small fiberglass boats (~ 6m in length) with outboard motors equipped with traps for lobster and semiautonomous hookah diving system for abalone [63]. Both abalone and lobster have minimum harvestable size limits and seasonal area closures [65] Furthermore, abalone fishery has yearly catch limits, established by the regional government entity [65]. Majority of this catch is destined for international markets [61, 63]. The cooperative actively engages with the regional government to monitor the conditions of the stock [63]. The cooperative has also developed conservation strategies such as the designation of protection areas for lobster [63].

The major challenges to the management of concessioned taxa are environmental change and illegal fishing. Impacts of heat waves and hypoxia related to El Niño, which resulted in a number of mass-mortality events of benthic taxa, require novel governance approaches [64, 66]. However, fishers from the area have expressed concerns over their ability to adequately adapt to the environmental changes that are occurring in this region [64]. Furthermore, threats from illegal fishing and poaching are especially acute, given that this region has the highest concentration of fishers in BCS [63]. This situation constrains the capacity of the cooperative to effectively monitor the use of its concession, which creates incentives for its members to break their own rules [67]. Cardenas Carpio [63] explains that some of these governance failures are rooted in serious violations to the democratic principles of cooperatives, characterized by flawed elections of the Board of Directors [63].

#### 3.5.3 Archetype III: Concessions of the Navachiste-Vinorama-Macapule lagoon complex

In the Navachiste-Vinorama-Macapule (NVM) lagoon complex of northern Sinaloa several cooperatives have an agreement to jointly manage their individual concessions for a specific resource, estuarine shrimp. Here, artisanal fishers mainly operate inside bays, coastal lagoons, and estuaries where small shrimp, of lower value than the one captured by industrial fishers, can be found [68, 69]. The fishery occurs in “pangas” (boats of 6 and 9 meters in length with 50–100 hp outboard motors) with a diversity of permitted gear including small trawling nets (“changos”), gill nets (“chinchorros de linea”), cast nets (“atarrayas”), and a traditional net called “suripera” which is a modified cast net that fishers drag along the floor using sailboats [69–72]. Fishers target mostly blue shrimp (*Litopenaeus stylirostris*), white shrimp (*Litopenaeus vannamei*), and brown shrimp (*Farfantepenaeus californiensis*), of which blue shrimp and white shrimp have the highest value in the market [68, 69]. The fishery is regulated by a seasonal closure with variable dates (usually from March to September) that aims to protect the reproduction and growth of shrimp. There are no quotas or minimum size limits [71, 72].

Due to the complexity of the landscape and the difficulty to forecast the movement patterns of shrimp, the cooperatives had to find a way to expand their area of work beyond their concessions and match their traditional fishing territories, which were historically defined by the areas of operation of the federations. Twenty-four fishing cooperatives from the NVM lagoon complex, which used to belong to a single federation, came up with a novel solution. Soon after they were granted concessions, in 1999, they signed two agreements. The first legal contract was an “conjoint agreement” (“*acuerdo de mancomún*”), where fishers agreed to share their fishing grounds with all other signing cooperatives, creating the “conjoint concession” (c*oncesion mancomunada*”) entity. In the second contract, “capture agreement” (“*acuerdo de captura”*), fishers agreed on a series of fishing regulations, particularly gear types. Nowadays five of the original 24 cooperatives belong to a different federation, but cooperatives still coordinate surveillance and lobbying efforts (e.g., towards federal and state agencies in charge of relevant fisheries programs and subsidies). Decisions of the “conjoint concession” are made through a committee where each cooperative is represented by its president, however, in contrast with the federations, this committee has no authority over the actions of the fishing cooperatives (Raul Leal Felix Pers. conv).

The biggest challenge for the implementation and management of conjoint concessions stems from the ongoing fragmentation of the federations of cooperatives. As more and more new federations are created, including many that harvest resources other than shrimp, development of collaborative initiatives among cooperatives becomes more challenging. Furthermore, the sustainable use of resources continues to be highly impacted by the lack of minimum size limits, illegal fishing practices, and impacts from inland activities that damage the mangrove system such as agriculture, deforestation, and aquaculture [71, 72].

#### 3.5.4 Archetype IV: *Concessions of the Tamiahua lagoon complex*

The Laguna de Tamiahua is a shallow, coastal lagoon located in the state of Veracruz, Mexico. According to Castañeda and Contreras (2001), Tamiahua Lagoon is the third largest coastal lagoon in Mexico and in 2005 it was designated as Ramsar site due to its mangrove forest, which is considered one of the best-structured and largest coastal forests located north of the Papaloapan River [73].

Eight fishing cooperatives (SCPPs) hold fishing concession titles for multiple species within the lagoon [73]. In 1995, these cooperatives signed a boundary agreement to define their operational areas and organize fishing activities within the lagoon [73]. These SCPPs include La Ribera de Tampico Alto, Pescadores de Cabo Rojo, La Huasteca Veracruzana, Ostioneros del Sur, Pescadores Unidos de la Reforma, Ostioneros de Saledero, Tamiahua, and Pescadores de Tamihua. Through their concession titles all these cooperatives have access to more than one species which can include oysters (*Crassostrea virginica*), various estuarine fish species, shrimp *(Farfantepenaeus aztecus* and *Litopenaeus setiferus*), and swimming crab (*Callinectes sapidus*). Oysters are the most significant fishery in terms of volume, followed by fish, shrimp, and swimming crab [73]. The fishing gear used by these SCPPs includes gillnet, seine net, cast net, gaff or rake, brass band, and swimming crab traps and hooks [73–75]

Despite the existent environmental regulations, the Laguna de Tamiahua faces significant challenges. The major issues that have been detected in the area comprise of the mangrove deforestation for construction, charcoal making and creating pastures, the process of eutrophication within the lagoon, pollution from various sources including garbage, wastewater, agrochemicals, thermal discharges, oil spills [75].

#### 3.5.5 Archetype V: *Sargassum concessions of Baja California*

The Sargassum concession of Baja California is registered to a company Agarmex S. A., founded in 1944 and located in Ensenada, Baja California. It is one of the three concessions in our database that are not given to fishing cooperatives. The company obtained its first concession in 1979 for the extraction of red algae (*Gelidium sp*. and *Gracilaria sp*.), which is used for the production of agar-agar, a jelly-like substance with a wide application in food as well as pharmaceutical and scientific industries [76, 77]. The current concession consists of 38 polygons distributed along the entire western coast of Baja California for the harvest of red sargassum (*Gelidium robustum*). The fishery is performed with a fiberglass boat (~ 6m in length) equipped with an outboard motor [78]. Algae is harvested using hookah diving, which consists of a diver connected to an air compressor and one to two deck hands and a boat captain [78]. Interestingly, fishing cooperatives are frequently subcontracted by the company during the harvesting season [78]. Once dried, the product is sold in international markets where it reaches high values (> 1,500 USD per metric ton).

The fishery has to follow the guidelines of a management plan for the state of Baja California (Plan de Manejo Pesquero de Algas Marinas de Baja California), which determines harvesting techniques, harvest volumes and harvest seasons, in order to avoid risking the biomass and algal diversity of the region [78]. The harvest has maintained relatively stable levels, at around 1000 metric tons of dry weight annually for the most important species, *Gelidium sp*, since the 1980s [78]. According to the estimates from a harvest in a period between 1985 and 1997, the resource was not overexploited [79]. However, the effects of climate change are likely to affect available biomass over a longer-term horizon either directly or through changes in community structure [79, 80].

## 4. Discussion

Previous studies of TURFs had mostly focused on a few successful case studies (e.g. [13–15, 57]). This is not surprising as successful stories are disproportionately represented in common pool and socio-ecological systems scientific literature [37, 81]. Limiting small-scale fisheries studies to successful case studies of fisheries management can result in the implementation of ineffective management strategies that may have adverse effects on both the environment and the livelihoods of fishing communities. This is due to the risk of overgeneralizing management approaches that may not be appropriate in different contexts [37]. It can also limit our understanding of the broader social, economic, and environmental factors that influence fisheries management effectiveness, as it ignores the challenges and failures [37, 81].

Our study is a step towards improving this situation by initiating the analysis of a management system in its entirety. Our dataset showed that TURFs are highly dynamic and heterogeneous entities that vary significantly depending on the social, economic, and environmental contexts in which they are implemented. The nature of existing management arrangements and their functioning is deeply rooted in the historical context within which they were formed and further shaped by existing social and ecological dynamics. Our study further revealed the need for a holistic research and management approach that considers the heterogeneity of TURFs and takes into account the broader social, economic, and environmental factors that influence their performance.

By mapping the Mexican system and creating a new dataset, we set the ground for a deep analysis of both successful and unsuccessful case studies in the future. In particular, the typology presented here can serve as a guideline to study other TURF systems globally and to expand our understanding of the conditions that lead to successful TURFs, and other tenure systems. The archetypes provide a way to simplify these systems by highlighting patterns and identifying key variables. This provides a starting point for the development of models that can predict how the system will respond to different inputs and perturbations. Moreover, as the characterization of these archetypes evolves, it can serve as a common language for researchers and practitioners across the world and facilitate cross-learning and collaboration.

Our analysis suggests that the Mexican concession landscape is much more extensive than previously thought and largely dominated by fishing cooperatives, which is likely the product of historical developments. Although concessions were first implemented for the benefit of private companies, they became a tool to protect and empower social organizations during the socialist and neoliberalists periods. The history of their development also determined their geographic distribution and explains the currently unequal spread of TURFs along the country and the diversity of systems in which they are present, from coral reefs and kelp forests to coastal lagoons and mangroves.

The evaluation of the archetypes in Mexico further revealed the polylithic nature of the TURF system. Archetypes I and II, which include large and mainly oceanic concessions, are the best-known cases from Mexico. A number of studies have shown that cooperatives from this archetype are capable of achieving sound social and ecological outcomes. Among the cooperatives from the Pacifico Norte region of Baja California Sur, a high social capital has led to sophisticated management strategies, such as voluntary no-take zones and the development of systematic monitoring programs [82]. However, there remain many understudied TURFs within these two archetypes, exposing the need for further studies.

Archetypes III and IV are likely the most numerous groups of TURFs and the one that has been poorly analyzed in previous research efforts. Novelty of this type is represented by voluntary arrangements for joint concession management. Interestingly, all these TURFs are present in lagoons and estuarine bodies that have been highly subdivided. As we will discuss later, these agreements for joint concessions might be playing an important role in coping with poor spatial TURF design and uncertainty in the distribution of the targeted species.

Lastly, archetype V is a sole representative of the private sector TURF system, which comprises 1.5% of all Mexican TURFs. Although presently sparse, our analysis of Mexico’s TURFs history showed that this was, in fact, the earliest model of TURFs in the country.

Our review of the Mexican TURF landscape made evident that although Mexico is home to many successful concessions, there is room for improvement. The long-term sustainability of a concession and its fisheries depends upon the legal, social, and ecological enabling conditions [83]. As with other catch share schemes, spatial and temporal design characteristics determine level of exclusivity in access and the performance of TURFs [84, 85]. Spatially, clarity on the TURF boundaries, as well as matching the spatial distribution of the targeted species, are key to achieve exclusivity in access and to facilitate enforcement [9, 83, 84]. Temporarily, for TURFs to operate successfully, they often need to provide security on access over long periods of time and offer a high probability of renewal [86]. Although these conditions seem to be present in some TURFs in Mexico, particularly successful case studies of archetypes I and II [13–15, 57, 59, 87], improvements are still necessary nationwide.

Spatially, the overlap in polygons, particularly in the state of Sinaloa, underscores a lack of clarity in TURF boundaries, which can lead to conflicts and disincentivize stewardship. Polygon overlap can create competition for resources between TURF owners and, without management agreements, lead to a race to fish and the over-exploitation of resources [84]. Furthermore, early concessions were large but frequently got subdivided as the number of cooperatives within a particular region grew. This led to some of the TURFs to be relatively small in size, compared to the dispersal capacity of the targeted species. In particular, the concessions that are located along the coast of Sinaloa, Chiapas, and Veracruz (Fig 1, Panels C, E, and F) that target mobile shrimp are relatively small, compared to the larger concessions that target other mobile species elsewhere in Mexico (i.e. Baja California Sur) (Fig 1, Panel B).

The mismatch between movement capacity of a targeted species and the size of TURF is a common problem in the implementation of spatial marine management tools [84, 88–91]. Two strategies emerge from previous studies that can help overcome social and ecological scale mismatches. The first one is to implement management tools that expand over large areas [84]. The second one, and far less studied, is the development of jointly managed concessions between two or more cooperatives [11, 92]. The “conjoint” or “*mancomunada*” TURFs in Mexico are an interesting new case study of inter-TURF cooperation that needs to be explored far more broadly. It would be important to assess how these agreements help cooperatives deal with uncertainty in resource distribution. During the conversation with our contact at the NVM system he explained that “it is better to make decisions during the closed season, that is when things are calm, because it’s then when nobody knows where the shrimp will be” (Raul Leal Felix Pers. conv). This exemplifies the significant weight that uncertainty in distribution plays on decision making and the important role that joint concessions might be having when environmental conditions lead to an uneven distribution of shrimp among TURF owners.

Lastly, although concessions in Mexico are relatively long lasting (5–20 years; DOF, 2018), the renewal process can be a considerable challenge. To get their concessions renewed, TURF owners need to pay for a socio-environmental study. The process can take up to three years, during which the cooperatives can’t legally enforce their areas. It is important to find ways in which to make the renewal process more efficient. For example, currently the renewal process does not take advantage of the catch records kept by these cooperatives, however many of these cooperatives are careful about keeping the catch records and those could be used as a measure of performance to speed up the renewal process.

Differences in the number of targeted species among Mexican TURFs also set the ground for interesting future research. In contrast with the TURFs located in the Yucatan peninsula (Quintana Roo and Yucatan states) where access rights are only granted for lobster, TURFs from Baja California Peninsula (Baja California and Baja California Sur), Veracruz and Chiapas are assigned for the exploitation of more than one species. Access to a large portfolio of species can provide several advantages. First it facilitates surveillance as it limits the number of species available for people outside of the owning cooperatives [83, 84]. Second, it promotes resilience since having a large portfolio can allow fishers to switch between targeted species and compensate for losses after market or environmental shocks [93]. However, the diversification of the targeted species portfolio can also lead to design challenges. Each targeted species may have a different area of distribution which may not perfectly overlap with the TURF area, a problem that has also been identified in Marine Protected Areas [84, 91, 94]. Therefore, two cooperatives could be sharing the same fishing ground leading to competitive behavior and overharvest [11, 88]. Second, each species has particular management requirements. Developing a management plan for all the species they have under concession can be a great challenge, as it has been for the SCPP Bahia Magdalena [64].

TURFs can provide many benefits as they promote long term sustainability and allow fishers to gain stewardship and authority over the management of their resources [64]. Further, past studies have shown that, when appropriately designed and managed, TURFs can lead to strong social arrangements and improve the function of community led fisheries institutions, such as cooperatives, ultimately leading to healthier resources [95, 96]. Mexico has a great opportunity in its TURF system that can be expanded and improved.

Our research project has laid a solid groundwork for conducting fruitful research, serving as an important initial step towards exploring the Mexican TURF landscape and building strong, resilient small-scale fisheries locally. To achieve this goal, it may be necessary to conduct a comparative case study to investigate the diversity within individual archetypes. With the current dataset, we can analyze the concessions’ spatial distribution, which can help evaluate the impacts of various environmental factors, including those resulting from climate change. Precisely, comprehending the spatial distribution of TURFs can facilitate the prediction of the potential effects of species distribution alterations. By augmenting this dataset with more information on each cooperative, including their governance structure, market strategies, and catch trends, we can acquire a better understanding of the factors that contribute to different performance levels. Furthermore, creating a robust documentation of the Mexican TURF system can inform the analysis and implementation of OECMs worldwide. Understanding the history, evolution, and performance of long-standing OCEM systems, such as Mexico’s, provides a unique opportunity for enhancing similar systems globally. Our study serves as a necessary starting point towards achieving this objective.

## Supporting information

S1 Table Outcome of the literature search in Google Scholar. The table includes terms searched, the number publications found, and the number of relevant publications used in this study.
(DOCX)

S2 Table Characteristics of key studies. Includes characteristics of key studies (N = 59) used to create a historical timeline and overview of Mexican concessions. List is arranged by geographical focus.
(DOCX)

S3 Table Taxa extracted from concession titles (N = 215). All concession titles report both a common name and a scientific name except for those that are marked in bold, which report only a common name. The total number of unique taxa (i.e., excluding duplicate scientific names or genera (e.g., *Panulirus spp*.) when species are listed (*Panulirus argus*, *Panulirus gracilis*, *Panulirus inflatus*, *Panulirus interruptus*) for which concessions have been issued is 26. Taxa in grey were not counted towards unique taxa.
(DOCX)
