# Supplementary material for: Sustaining small-scale fisheries through a nation-wide Territorial Use Rights in Fisheries system
Source: PLoS One. 2023 Jun 27;18(6):e0286739. doi: 10.1371/journal.pone.0286739 (PMC10298796; doi:10.1371/journal.pone.0286739)
Supplement: S1 Table — The table includes terms searched, the number publications found, and the number of relevant publications used in this study. (DOCX) [file pone.0286739.s001.docx]

S1 Table. Outcome of the literature search in Google Scholar. The table includes terms searched, the number publications found, and the number of relevant publications used in this study.

| **SEARCH TERM** | **TOTAL PUBLICATIONS** | **RELEVANT PUBLICATIONS** |
| --- | --- | --- |
| Mexico pesca "concesiones territoriales" | 187 | 47 |
| Mexico pesca "concesión territorial" | 74 | 35 |
| Mexico “concesiones pesqueras” | 85 | 58 |
| Mexico “concesión pesquera” | 46 | 25 |
| Mexico “fishing concessions” | 254 | 85 |
| Mexico “fishing concession” | 148 | 78 |
| Mexico “territorial use rights” | 870 | 76 |
