# Supplementary material for: Sustaining small-scale fisheries through a nation-wide Territorial Use Rights in Fisheries system
Source: PLoS One. 2023 Jun 27;18(6):e0286739. doi: 10.1371/journal.pone.0286739 (PMC10298796; doi:10.1371/journal.pone.0286739)
Supplement: S2 Table — Includes characteristics of key studies (N = 59) used to create a historical timeline and overview of Mexican concessions. List is arranged by geographical focus. (DOCX) [file pone.0286739.s002.docx]

S2 Table. Characteristics of key studies. Includes characteristics of key studies (N = 59) used to create a historical timeline and overview of Mexican concessions. List is arranged by geographical focus.

| **AUTHORS** | **TITLE** | **PUBLISHER** | **YEAR** | **PUBLICATION TYPE** | **GEOGRAPHICAL FOCUS** | **INSTITUTIONAL FOCUS** |
| --- | --- | --- | --- | --- | --- | --- |
| Arafeh Dalmau, Nur; | Diseño de una red de Áreas Marinas Protegidas en un contexto transfronterizo: Enlazando Baja California con las Áreas Marinas Protegidas de California | Universidad Autónoma de Baja California | 2016 | master thesis | Baja California | NA |
| Arafeh-Dalmau, Nur; Torres-Moye, Guillermo; Seingier, Georges; Montaño-Moctezuma, Gabriela; Micheli, Fiorenza; | Marine spatial planning in a transboundary context: linking Baja California with California's network of marine protected areas | Frontiers in Marine Science | 2017 | peer reviewed journal | Baja California | NA |
| Delgado Ramírez, Claudia E; Soto Aguirre, Enrique; | Co-manejo pesquero e innovación social: el caso de la pesquería de erizo rojo (Strongylocentrotus franciscanus) en Baja California | Sociedad y Ambiente | 2018 | peer reviewed journal | Baja California | Concession of SCPP Buzos y Pescadores del Ejido Coronel Esteban Cantú |
| Ramírez-Félix, Evlin; Manzo-Monroy, Héctor G; | Comparación entre el uso de dos derechos de acceso pesquero, concesiones y permisos, en la pesquería de erizo rojo de mar, Strongylocentrotus franciscanus (Agassiz), en Santo Tomás, Baja California, México / Modelling the use of two fishery access rights, concessions and licences, in the red sea urchin, Strongylocentrotus franciscanus (Agassiz), fishery at Santo Tomás, Baja California, Mexico | Ciencias Marinas | 2004 | peer reviewed journal | Baja California | Concession adjacent to the community of Santo Tomas |
| Sánchez, Federico Alfonso Méndez; | Co-management and Small-scale Fisheries in Mexico: The Case of a Fishers' Cooperative in Cedros and San Benito Islands | University of Auckland | 2012 | master thesis | Baja California | Concession of SCPP Pescadores Nacionales de Abulón |
| Tamayo, David; | The Perilous Borderlands: The Role of Anti-Japanese Hysteria in American Efforts to Annex Baja California, 1900–1942 | California History | 2020 | peer reviewed journal | Baja California; Baja California Sur | Abalone concession granted to a Japnese company |
| Álvarez, Pablo; Espejel, Ileana; Bocco, Gerardo; Cariño, Micheline; Seingier, Georges; | Environmental history of Mexican North Pacific fishing communities | Ocean & Coastal Management | 2018 | peer reviewed journal | Baja California Sur (Northern Pacific region) | Concessions of cooperatives from the Northern Pacific (Pacifico Norte) region of Baja California Sur |
| Bautista González, Karina A; | Análisis de vulnerabilidad de los manglares de la costa occidental de Baja California Sur, México, con miras a identificar acciones de adaptacion local | CATIE, Turrialba (Costa Rica) | 2014 | master thesis | Baja California Sur | NA |
| Cariño, Micheline; González, Luis Alberto; Castro, Erín; Ojeda, Esteban; | Vieja y nuevas concepciones de la frontera: aportes teóricos y reflexiones sobre la historia sudcaliforniana | Estudios Fronterizos | 2000 | peer reviewed journal | Baja California Sur | Historical evaluation of pearl concessions |
| Cariño, Micheline; Monteforte, Mario; | Las perlas y los hombres en el Golfo de California, 1500-2005 | Pscadores en América Latina y el Caribe: Espacio, población, producción y política | 2011 | book chapter | Baja California Sur | Historical evaluation of pearl concessions |
| Cariño, Micheline; Monteforte, Mario; | Las minas marinas del Golfo de California: del extractivismo a la sustentabilidad | Relaciones. Estudios de Historia y Sociedad | 2018 | peer reviewed journal | Baja California Sur | Historical evaluation of pearl concessions |
| Cunningham, Erica; Apel, Ashley; McIlwain, Karly; Van Leuvan, Tonya; | Mexican Baja California FEDECOOP Benthic Species Territorial Use Rights for Fishing System | Environmental Defense Fund | 2013 | report | Baja California Sur (Northern Pacific region) | Concessions of cooperatives from the Northern Pacific (Pacifico Norte) region of Baja California Sur |
| Guerrero, Jose Manuel Crespo; Pelcastre, Araceli Jimenez; | Arrangement of the riverside commercial fishing sector in the Biosphere Reserve El Vizcaino (Mexico)/Organizacion del sector pesquero comercial ribereno en la Reserva de la Biosfera El Vizcaino (Mexico) | Revista Geográfica Venezolana | 2016 | peer reviewed journal | Baja California Sur (Northern Pacific region) | Concessions of cooperatives from the Northern Pacific (Pacifico Norte) region of Baja California Sur |
| Hernández-Padilla, Juan Carlos; Navarro-González, Jessica Adriana; Salcido-Guevara, Luis A; Sánchez-Cárdenas, Rebeca; Gutiérrez-González, José Luis; Yee-Duarte, Josué Alonso; | Variación espacio-temporal en la densidad y estructura de tallas de Megastraea undosa (Gastropoda: Turbinidae) en la costa occidental de Baja California Sur, México | Revista de Biología Marina y Oceanografía | 2021 | peer reviewed journal | Baja California Sur (Northern Pacific region) | Concessions of cooperatives from the Northern Pacific (Pacifico Norte) region of Baja California Sur |
| McCay, Bonnie J; | Territorial use rights in fisheries of the northern Pacific coast of Mexico | Bulletin of Marine Science | 2017 | peer reviewed journal | Baja California Sur (Northern Pacific region) | Concessions of cooperatives from the Northern Pacific (Pacifico Norte) region of Baja California Sur |
| McCay, Bonnie J; Micheli, Fiorenza; Ponce-Díaz, Germán; Murray, Grant; Shester, Geoff; Ramirez-Sanchez, Saudiel; Weisman, Wendy; | Cooperatives, concessions, and co-management on the Pacific coast of Mexico | Marine Policy | 2014 | peer reviewed journal | Baja California Sur (Northern Pacific region) | Concessions of cooperatives from the Northern Pacific (Pacifico Norte) region of Baja California Sur |
| Nomura, Keiko; | Fisheries Connectivity Networks to Measure the Adaptive Capacity of Small-Scale Fisheries in the Baja California Peninsula, Mexico | Oregon State University | 2020 | master thesis | Baja California Sur | NA |
| Olvera, Martha Micheline Cariño; Monteforte, Mario; | Oasis entre mares: Historiografía ambiental de Baja California Sur | La historia ambiental en México: Estudios de caso | 2019 | book chapter | Baja California Sur | Historical evaluation of pearl concessions |
| Olvera, Martha Micheline Cariño; Sánchez, Mario Monteforte; | La historiografía ambiental sudcaliforniana y la sustentabilidad local | Historia Ambiental Latinoamericana y Caribeña | 2018 | peer reviewed journal | Baja California Sur | Historical evaluation of pearl concessions |
| Phillips, Bruce; Bourillón, Luis; Ramade, Mario; | Case study 2: the Baja California, Mexico, lobster fishery | Seafood ecolabelling: Principles and practice | 2008 | book chapter | Baja California Sur (Northern Pacific region) | Concessions of cooperatives from the Northern Pacific (Pacifico Norte) region of Baja California Sur |
| Quintana, Anastasia CE; | A Spark for Collective Action: Challenges and Opportunities for Self-Governance in Temporary Fisher-Designed Fish Refuges in Mexico | Duke University | 2020 | doctoral thesis | Baja California Sur (El Corredor region) | Evaluation of fisheries refugia |
| Quintana, Anastasia CE; Basurto, Xavier; | Community‐based conservation strategies to end open access: The case of Fish Refuges in Mexico | Conservation Science and Practice | 2021 | peer reviewed journal | Baja California Sur (El Corredor region) | Evaluation of fisheries refugia |
| Santos, Antonio Ortega; | Extractivismo marino-colonial. Apropiación asimétrica de recursos marinos en el golfo de California (México) siglos XVI-XXI | Relaciones Internacionales | 2021 | peer reviewed journal | Baja California Sur | Historical evaluation of pearl concessions |
| Torre, Jorge; Fernández Rivera-Melo, Francisco; | Acción sin daño: un análisis de las intervenciones de una organización de la sociedad civil ambientalista en comunidades costeras del Noroeste de México | Relaciones. Estudios de Historia y Sociedad | 2018 | peer reviewed journal | Baja California Sur (Northern Pacific region) | Concessions adjacent to Magdalena Island and Natividad Island |
| WinklerPrins, Antoinette; Alvarez, Pablo; Bocco, Gerardo; Espejel, Ileana; | From Ranching to Fishing–The Cultural Landscape of the Northern Pacific Coast of Baja California, Mexico | Focus on Geography | 2016 | peer reviewed journal | Baja California Sur (Northern Pacific region) | Concessions of cooperatives from the Northern Pacific (Pacifico Norte) region of Baja California Sur |
| Young, Emily; | State intervention and abuse of the commons: fisheries development in Baja California Sur, Mexico | Annals of the Association of American Geographers | 2001 | peer reviewed journal | Baja California Sur | NA |
| Solano, Neyra; Lopez-Ercilla, Ines; Fernandez-Rivera Melo, Francisco J; Torre, Jorge; | Unveiling women’s roles and inclusion in Mexican small-scale fisheries (SSF) | Frontiers in Marine Science | 2021 | peer reviewed journal | Baja California Sur; Quintana Roo | NA |
| Solano, Neyra; Lopez-Ercilla, Ines; Fernandez—Rivera Melo, FJ; Torre, Jorge; | Revelando el papel de la mujer y su inclusión en la Pesca en Pequeña Escala (PPE) mexicana | Frontiers in Marine Science | 2020 | peer reviewed journal | Baja California Sur; Quintana Roo | NA |
| Villaseñor-Derbez, Juan Carlos; Aceves-Bueno, Eréndira; Fulton, Stuart; Suarez, Alvin; Hernández-Velasco, Arturo; Torre, Jorge; Micheli, Fiorenza; | An interdisciplinary evaluation of community-based TURF-reserves | PloS one | 2019 | peer reviewed journal | Baja California Sur (Northern Pacific region); Quintana Roo (Sian Ka'an Biosphere Reserve region) | Concessions of SCPP Buzos y Pescadores de la Baja California, SCPP Cozumel, and SCPP José María Azcorra |
| Álvarez, Pablo; Delgado, Claudia; Seingier, Georges; Espejel, Ileana; | Historia Ambiental del comanejo adaptativo en dos regiones pesqueras del noroeste mexicano | Relaciones. Estudios de Historia y Sociedad | 2018 | peer reviewed journal | Baja California; Baja California Sur (Northern Pacific region) | Concessions of SCPP Buzos y Pescadores del Ejido Coronel Esteban Cantú, and of cooperatives from the Norther Pacific (Pacifico Norte) region |
| Aceves-Bueno, Eréndira; Cornejo-Donoso, Jorge; Miller, Steve J; Gaines, Steven D; | Are territorial use rights in fisheries (TURFs) sufficiently large? | Marine Policy | 2017 | peer reviewed journal | International; Baja California Sur (Northern Pacific region) | Concessions of cooperatives from the Northern Pacific (Pacifico Norte) region of Baja California Sur |
| Tholan, Brittany; | A Global Database of Tenure and Access Rights for Small-Scale Fisheries: A Preliminary Assessment | Duke University | 2022 | master thesis | International; Baja California Sur (Northern Pacific region) | Concessions of cooperatives from the Northern Pacific (Pacifico Norte) region of Baja California Sur |
| Viana, Daniel F; Gelcich, Stefan; Aceves‐Bueno, Erendira; Twohey, Becky; Gaines, Steven D; | Design trade‐offs in rights‐based management of small‐scale fisheries | Conservation Biology | 2019 | peer reviewed journal | International; Mexico wide | NA |
| Cinner, JE; Basurto, Xavier; Fidelman, Pedro; Kuange, John; Lahari, Rachael; Mukminin, Ahmad; | Institutional designs of customary fisheries management arrangements in Indonesia, Papua New Guinea, and Mexico | Marine Policy | 2012 | peer reviewed journal | International; Sonora (Tiburon Island region) | Concession of Seri* |
| Liu, Jing; Qin, Tianbao; | A comparative analysis of fishing rights from a transaction cost perspective | Ecological Economics | 2018 | peer reviewed journal | International; Sonora (Tiburon Island region) | Concession of Seri* |
| García, Milton Gabriel Hernández; | Propuestas para la revitalización de la pesca ribereña y la conservación de los ecosistemas costeros en México | Revista de Geografía Agrícola | 2020 | peer reviewed journal | Mexico wide | NA |
| Aceves‐Bueno, Eréndira; Miller, Steve J; Cornejo‐Donoso, Jorge; Gaines, Steven D; | Cooperation as a solution to shared resources in territorial use rights in fisheries | Ecological Applications | 2020 | peer reviewed journal | Quintana Roo (Sian Ka'an Biosphere Reserve region) | Concession of SCPP Pescadores de Vigía Chico |
| Brenner, Ludger; Vargas del Río, David; | Gobernabilidad y gobernanza ambiental en México: La experiencia de la Reserva de la Biosfera Sian Ka'an | Polis | 2010 | peer reviewed journal | Quintana Roo (Sian Ka'an Biosphere Reserve region) | Concession of SCPP Pescadores de Vigía Chico |
| Cochran, Kathryn A; | Fishery Co-Management: The Case Of Punta Allen Spiny Lobster Fishery | Agricultural and Applied Economics Association | 1998 | conference proceedings | Quintana Roo | Concession of SCPP Pescadores de Vigía Chico |
| Cunningham, Erica; Apel, Ashley; Ruiter, Pam; Van Leuvan, Tonya; | Mexican Vigía Chico Cooperative spiny lobster territorial use rights for fishing program | Environmental Defense Fund | 2013 | report | Quintana Roo (Sian Ka'an Biosphere Reserve region) | Concession of SCPP Pescadores de Vigía Chico |
| Fulton, Stuart; Caamal, Jacobo; Ribot, Constanza; Lucas, Beatriz; Garcia, Citlli; Bourillon, Luis; Flores, Eglé; | Coral reef monitoring with fishers’ participation in Quintana Roo, Mexico: building social capital to preserve marine ecosystems | Gulf and Caribbean Fisheries Institute | 2013 | conference proceedings | Quintana Roo (Sian Ka'an Biosphere Reserve and Banco Chinchorro regions) | Concessions of SCPP Cozumel, SCPP José María Azcorra, SCPP Langosteros del Caribe, SCPP Andrés Quintana Roo, and SCPP Banco Chinchorro |
| Méndez-Medina, Crisol; Schmook, B; Basurto, X; Urrea-Mariño, U; Alcalá, G; | Comités comunitarios en Sian Ka’an: redes de colaboración para enfrentar los efectos del cambio climático | Pescadores en México y Cuba: Retos y oportunidades ante el cambio climático | 2020 | book chapter | Quintana Roo (Sian Ka'an Biosphere Reserve region) | Concessions of SCPP Pescadores de Vigía Chico, SCPP Cozumel, and SCPP José María Azcorra |
| Méndez-Medina, Crisol; Schmook, Birgit; Basurto, Xavier; Fulton, Stuart; Espinoza-Tenorio, Alejandro; | Achieving coordination of decentralized fisheries governance through collaborative arrangements: A case study of the Sian Ka'an Biosphere Reserve in Mexico | Marine Policy | 2020 | peer reviewed journal | Quintana Roo (Sian Ka'an Biosphere Reserve region) | Concessions of SCPP Pescadores de Vigía Chico, SCPP Cozumel, and SCPP José María Azcorra |
| Méndez-Medina, Crisol; Schmook, Birgit; McCandless, Susannah R; | The Punta Allen cooperative as an emblematic example of a sustainable small-scale fishery in the Mexican Caribbean | Maritime Studies | 2015 | peer reviewed journal | Quintana Roo (Sian Ka'an Biosphere Reserve region) | Concession of SCPP Pescadores de Vigía Chico |
| Moreno, Andrea; Bourillón, Luis; Flores, Eglé; Fulton, Stuart; | Fostering fisheries management efficiency through collaboration networks: the case of the Kanan Kay Alliance in the Mexican Caribbean | Bulletin of Marine Science | 2017 | peer reviewed journal | Quintana Roo (Sian Ka'an Biosphere Reserve and Banco Chinchorro region) | Concessions of SCPP Cozumel, SCPP José María Azcorra, SCPP Tulum, SCPP Langosteros del Caribe, SCPP Andrés Quintana Roo, and SCPP Banco Chinchorro |
| Velez, Mariana; Adlerstein, Sara; Wondolleck, Julia; | Fishers' perceptions, facilitating factors and challenges of community-based no-take zones in the Sian Ka’an Biosphere Reserve, Quintana Roo, Mexico | Marine Policy | 2014 | peer reviewed journal | Quintana Roo (Sian Ka'an Biosphere Reserve region) | Concessions of SCPP Pescadores de Vigía Chico, SCPP Cozumel, and SCPP José María Azcorra |
| Villanueva-Poot, Raúl; Seijo, Juan Carlos; Headley, Maren; Arce, Ana Minerva; Sosa-Cordero, Eloy; Lluch-Cota, Daniel Bernardo; | Distributional performance of a territorial use rights and co-managed small-scale fishery | Fisheries Research | 2017 | peer reviewed journal | Quintana Roo (Sian Ka'an Biosphere Reserve region) | Concession of SCPP Pescadores de Vigía Chico |
| Villanueva, Raul; Lara, Oswaldo Huchim; Seijo, Juan Carlos; Palomo, Leopoldo; Duarte, José A; | Distributional performance of two different rights-based managed small-scale lobster fisheries: Individual and collective territorial use rights regimes | Ocean & Coastal Management | 2019 | peer reviewed journal | Quintana Roo (Sian Ka'an Biosphere Reserve region) | Concessions of SCPP Pescadores de Vigía Chico, SCPP San Felipe, and SCPP Rio Lagartos |
| Alarcón, Rigoberto Arturo; | Las cooperativas pesqueras de los esteros del Sur de Sinaloa 1929-1980 | Anuario del Centro de Estudios Históricos Profesor Carlos SA Segreti | 2015 | peer reviewed journal | Sinaloa (Southern Sinaloa region) | Concessions of cooperatives from Southern Sinaloa |
| Basurto, Xavier; | How locally designed access and use controls can prevent the tragedy of the commons in a Mexican small-scale fishing community | Society and Natural Resources | 2005 | peer reviewed journal | Sonora (Tiburon Island region) | Concession of Seri* |
| Basurto, Xavier; | Biological and ecological mechanisms supporting marine self-governance: the Seri callo de hacha fishery in Mexico | Ecology and Society | 2008 | peer reviewed journal | Sonora (Tiburon Island region) | Concession of Seri* |
| Basurto, Xavier; Cinti, Ana; Bourillón, Luis; Rojo, Mario; Torre, Jorge; Weaver, A Hudson; | The emergence of access controls in small-scale fishing commons: A comparative analysis of individual licenses and common property-rights in two Mexican communities | Human Ecology | 2012 | peer reviewed journal | Sonora (Tiburon Island region) | Concession of Seri* |
| Basurto, Xavier; Gelcich, Stefan; Ostrom, Elinor; | The social–ecological system framework as a knowledge classificatory system for benthic small-scale fisheries | Global Environmental Change | 2013 | peer reviewed journal | Sonora (Tiburon Island region) | Concession of Seri* |
| Basurto, Xavier; Lozano, Alejandro García; | Commoning and the commons as more-than-resourcesA historical perspective on Comcáac or Seri fishing | Making Commons Dynamic | 2021 | book chapter | Sonora (Tiburon Island region) | Concession of Seri* |
| Bourillón-Moreno, Luis; | Exclusive fishing zone as a strategy for managing fishery resources by the Seri Indians, Gulf of California, Mexico | The University of Arizona | 2002 | doctoral thesis | Sonora (Tiburon Island region) | Concession of Seri* |
| Cudney-Bueno, Richard; Basurto, Xavier; | Lack of cross-scale linkages reduces robustness of community-based fisheries management | PloS one | 2009 | peer reviewed journal | Sonora (Northern Sonora region) | Concession of cooperative from Puerto Peñasco |
| Cudney-Bueno, Richard; Bourillón, Luis; Sáenz-Arroyo, Andrea; Torre-Cosío, Jorge; Turk-Boyer, Peggy; Shaw, William W; | Governance and effects of marine reserves in the Gulf of California, Mexico | Ocean & Coastal Management | 2009 | peer reviewed journal | Sonora (Northern Sonora region) | Concession of cooperative from Puerto Peñasco |
| Bennett, Abigail; | The influence of neoliberalization on the success and failure of fishing cooperatives in contemporary small-scale fishing communities: A case study from Yucatán, Mexico | Marine Policy | 2017 | peer reviewed journal | Yucatan | Concessions of various cooperatives from Yucatan |
| Bennett, Abigail; | Strengthening Small-Scale Fishing Cooperatives through Rights-Based Fisheries Management: A Case Study from Yucatán, Mexico | North American Association of Fisheries Economists | 2017 | conference proceedings | Yucatan | Concessions of various cooperatives from Yucatan |

*The concession granted to Seri or the Comcáac indigenous group of Sonora was established in 1975 by presidential decree and includes waters adjacent to the Tiburon Island. The remaining concessions were granted by CONAPESCA.
