# Supplementary material for: Sustaining small-scale fisheries through a nation-wide Territorial Use Rights in Fisheries system
Source: PLoS One. 2023 Jun 27;18(6):e0286739. doi: 10.1371/journal.pone.0286739 (PMC10298796; doi:10.1371/journal.pone.0286739)
Supplement: S3 Table — All concession titles report both a common name and a scientific name except for those that are marked in bold, which report only a common name. The total number of unique taxa (i.e., excluding duplicate scientific names or genera (e.g., Panulirus spp.) when species are listed (Panulirus argus, Panulirus gracilis, Panulirus inflatus, Panulirus interruptus) for which concessions have been issued is 26. Taxa in grey were not counted towards unique taxa. (DOCX) [file pone.0286739.s003.docx]

S3 Table. Taxa extracted from concession titles (N = 215). All concession titles report both a common name and a scientific name except for those that are marked in bold, which report only a common name. The total number of unique taxa (i.e., excluding duplicate scientific names or genera (e.g., *Panulirus spp.*) when species are listed (*Panulirus argus, Panulirus gracilis, Panulirus inflatus, Panulirus interruptus*) for which concessions have been issued is 26. Taxa in grey were not counted towards unique taxa.

| **Common name (Spanish)** | **Common name (English)** | **Scientific name** | **Mobility** | **Number of concessions** |
| --- | --- | --- | --- | --- |
| Escama estuarina | Estuarine finfish | **Actinopterygii** | demersal, reef-associated, pelagic; mobile | 14 |
| Escama general | Finfish | **Actinopterygii** | demersal, reef-associated, pelagic; mobile | 5 |
| Almeja pata de mula | Pustulose ark | ***Anadara spp*** | benthic sessile | 2 |
| Almeja catarina | Catarina scallop | ***Argopecten circularis*** | benthic sessile | 1 |
| Cangrejo | Crab | **Brachyura** | bentic mobile | 3 |
| Jaiba | Swimming crab | *Callinectes spp* | bentic mobile | 6 |
| Cangrejo azul | Blue crab | *Cardisoma guanhumi* | bentic mobile | 2 |
| Ostion | Oyster | ***Crassostrea spp*** | benthic sessile | 1 |
| Ostion de roca | Rock oyster | *Crassostrea iridescens* | benthic sessile | 3 |
| Ostion americano | Eastern oyster | *Crassostrea virginica* | benthic sessile | 8 |
| Algas marinas | Marine algae | *Gelidium spp* | benthic sessile | 1 |
| Sargazo rojo | Red algae | *Gelidium robustum* | benthic sessile | 4 |
| Abulon | Abalone | *Haliotis spp* | bentic mobile | 9 |
| Abulon amarillo | Pink abalone | *Haliotis corrugata* | bentic mobile | 2 |
| Abulon azul | Green abalone | *Haliotis flugens* | bentic mobile | 2 |
| Pepino de mar | Sea cucumber | ***Isostichopus spp*** | bentic mobile | 3 |
| Camaron azul | Pacific blue shrimp | *Litopenaeus stylirostris* | bentic mobile | 8 |
| Acamaya | River shrimp | *Macrobrachium spp* | bentic mobile | 2 |
| Langostino | Freshwater shrimp | *Macrobrachium carcinus* | bentic mobile | 1 |
| Caracol panocha | Wavy turban | ***Megastraea undosa*** | bentic mobile | 6 |
| Caracol burro | Pacific crown conch | *Melongena patula* | bentic mobile | 1 |
| Pulpo | Octopus | ***Octopus spp*** | bentic mobile | 2 |
| Langosta | Lobster | *Panulirus spp* | bentic mobile | 6 |
| Langosta del Caribe | Caribbean spiny lobster | *Panulirus argus* | bentic mobile | 13 |
| Langosta verde | Green spiny lobster | *Panulirus gracilis* | bentic mobile | 1 |
| Langosta azul | Blue spiny lobster | *Panulirus inflatus* | bentic mobile | 4 |
| Langosta roja | California spiny lobster | *Panulirus interruptus* | bentic mobile | 4 |
| Camaron | Shrimp | *Penaeus spp* | bentic mobile | 12 |
| Camaron de estero | Estuarine shrimp | *Penaeus spp* | bentic mobile | 144 |
| Camaron cafe | Brown shrimp | *Penaeus aztecus* | bentic mobile | 9 |
| Camaron blanco | Pacific white shrimp | *Penaeus vannamei* | bentic mobile | 8 |
| Erizo rojo | Red sea urchin | *Strongylocentrotus franciscanus* | bentic mobile | 2 |
| Erizo morado | Pacific purple sea urchin | *Strongylocentrotus pupuratus* | bentic mobile | 2 |
| Almeja pismo | Pismo clam | *Tlvela stuitorum* | benthic sessile | 5 |
